# Decision Thresholds for Medical Tests Under Ambiguity Aversion

**DOI:** 10.3389/frhs.2022.825315

**Published:** 2022-03-21

**Authors:** Dilek Sevim, Stefan Felder

**Affiliations:** ^1^Faculty of Business and Economics, University of Basel, Basel, Switzerland; ^2^CINCH, University of Duisburg-Essen, Essen, Germany

**Keywords:** ambiguity aversion, diagnostic ambiguity, therapeutic ambiguity, medical decision thresholds, demand for medical tests, value of information

## Abstract

We consider medical decision-making under diagnostic and therapeutic uncertainty and analyze how ambiguity aversion affects the decisions to test and treat, thereby contributing to the understanding of the observed heterogeneity of such decisions. We show that under diagnostic ambiguity (i.e., the probability of disease is ambiguous), prior testing becomes more attractive if the default option is no treatment and less so if the default option is treatment. Conversely, with therapeutic ambiguity (i.e., the probability of a successful treatment is ambiguous), ambiguity aversion reduces the tolerance toward treatment failure so that the test option is chosen at a lower probability of failure. We differentiate between conditional and unconditional ambiguity aversion and show that this differentiation has implications for the propensity to test. We conclude by discussing the normative scope of ambiguity aversion for the recommendations and decisions of regulatory bodies.

## Introduction

In medical practice, decisions are often based on limited information. Examples include situations in which data are either incomplete or not representative of the patient population so that the prevalence of a certain disease or the probability of success of a specific treatment cannot be unambiguously determined. Ambiguity in this sense differs from the risk, which refers to an objectively known probability distribution (1). The attitudes of decision makers (DMs) toward ambiguity about the correct probability of disease and treatment success across patients are relevant for their decisions. If they are ambiguity neutral, they will use a weighted average of the different probabilities available in the literature. However, starting with Ellsberg (2), a large body of literature has shown that people are not neutral about ambiguity but rather dislike it and are ambiguity averse. Evidence for ambiguity-averse behavior in the health context includes Curley et al. (3, 4), Ritov and Baron (5), Viscusi et al. (6), Viscusi and Magat (7), Gerrity et al. (8), and Portnoy et al. (9)^1^. Analyzing the effect of ambiguity aversion appears to be important for understanding variations in testing and treatment practice.

We use the smooth ambiguity model of Klibanoff et al. (13) (hereafter KMM) to study medical decisions. KMM define that ambiguity-averse DMs are more strongly averse to the uncertainty over the “right” probability than to the risk in lotteries with known probabilities. Analogous to risk aversion, they characterize ambiguity aversion, with a concave utility function defined over the space of expected utilities of lotteries. KMM also define the degree of ambiguity aversion of the DM according to the Arrow–Pratt coefficient of absolute risk aversion (13, 14). They present a two-stage model in which the expected utilities conditional on the possible probability distributions are computed using a von Neumann–Morgenstern utility function (characterizing risk aversion) in the first stage. In the second stage, these expected utilities are weighted with respect to the subjective probabilities of the DM using an increasing concave function (characterizing ambiguity aversion). An advantage of this approach is that a simple adaptation of the expected utility framework is sufficient for computations; the only difference is the utility functions applied at each stage.

This paper analyzes the effect of ambiguity aversion on decision thresholds if a diagnostic test is available to support the treatment decision. There are then two thresholds that mark the probability at which the DM starts to test (i.e., the testing threshold) and the probability at which they stop and treat directly (i.e., the test-treatment threshold). Our analysis is based on the two classical decision-making models under uncertainty: Pauker and Kassirer's model (15) on diagnostic risk and Eeckhoudt's and Viscusi model (16) on therapeutic risk. Eeckhoudt noted early on that in the two models, risk aversion has opposite effects on the threshold at which DMs are indifferent between treatment and no treatment. While the propensity of risk-averse DMs to treat increases under diagnostic risk, it decreases under therapeutic risk. The intuition for this result is that treatment decreases the spread of possible health states under diagnostic risk and increases it under therapeutic risk. Berger et al. (17) analyzed the effect of ambiguity aversion on the treatment decision and showed that it reinforces the effect of risk aversion^2^. Here, the spread of expected utility with and without treatment is decisive. Under diagnostic ambiguity, the treatment decreases the spread of expected utility, while under therapeutic ambiguity, it increases it. Again, treatment becomes more attractive under diagnostic ambiguity and less so under therapeutic ambiguity.

In our analysis, which additionally includes a test option, we focus on the effect of ambiguity aversion on the test and test-treatment thresholds and show that these thresholds shift in favor of treatment under diagnostic ambiguity and in favor of no treatment under therapeutic ambiguity. The propensity to test is determined by the difference between the two thresholds in the two settings. Because ambiguity aversion shifts the testing interval without necessarily narrowing or widening it, its effect on the propensity to test cannot be signed. Results on the demand for medical tests under diagnostic ambiguity have been inconsistent in the literature. Snow (19) found information that reduces or resolves ambiguity to always be valuable to ambiguity-averse individuals and that this value increases with the degree of ambiguity aversion. The demand for tests should therefore increase with the degree of ambiguity aversion. In contrast, Hoy et al. (20) explain the low take-up rates of genetic tests observed with ambiguity aversion. From an ex ante perspective, ambiguity-averse patients shy away from testing because it introduces uncertainty related to the different possible test outcomes. In order to elaborate on these contradictory results, we also address an alternative specification of ambiguity aversion that focuses not on the probabilities of possible events but rather on the ex ante evaluation of possible test outcomes. We show that under the latter approach, the propensity to test decreases under both diagnostic and therapeutic ambiguity.

The structure of the paper is as follows. In Section Decision Thresholds Under Diagnostic Ambiguity, we present medical decision-making under diagnostic ambiguity. In Section Decision Thresholds Under Therapeutic Ambiguity, we address therapeutic ambiguity. In order to simplify the presentation, we assume that the probabilities of disease (in the diagnostic model) and of treatment failure (in the therapeutic model) take two values only. In the Appendix, we provide formal proofs of the results for the general case of *n* > 2 different probabilities. In Section Discussion, we discuss the main findings, including the results for the alternative specification of ambiguity aversion and their implications from a social welfare perspective. In Section Conclusion, we summarize our findings.

## Decision Thresholds Under Diagnostic Ambiguity

**Figure 1:**
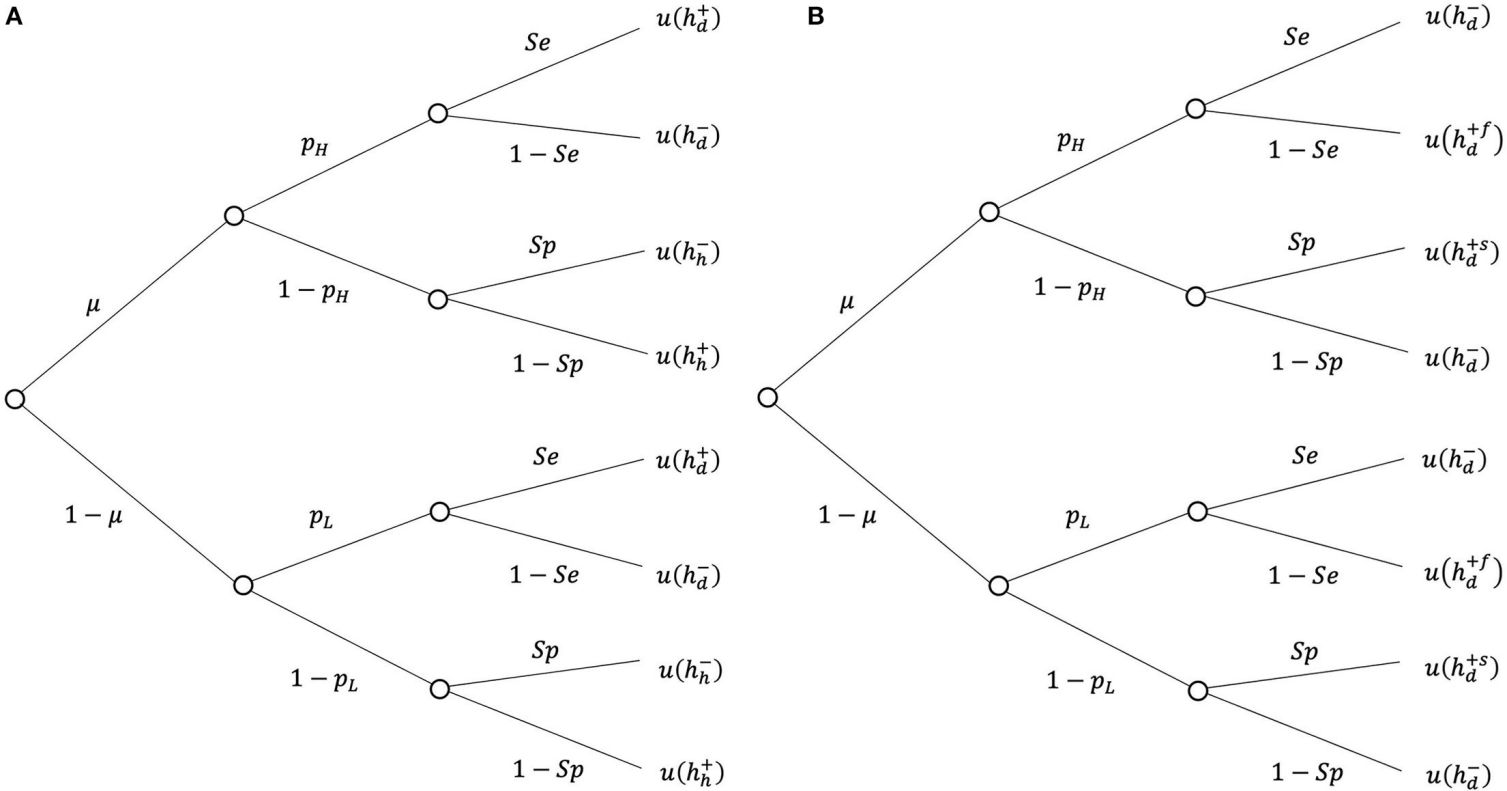
Decision tree of a test **(A)** diagnostic uncertainty; **(B)** therapeutic uncertainty.

Consider a situation in which a physician decides on the treatment of a patient with certain symptoms. The DM is uncertain about the patient's true state of health. Assume that there are two possible health states, healthy (*h*) and diseased (*d*), as well as three possible actions, treatment (+), no treatment (−), and diagnostic testing (*t*), followed by the treatment decision according to the test outcome, i.e., treatment if + and no treatment if −. The test correctly detects a sick patient with probability *Se* (sensitivity) and a healthy patient with probability *Sp* (specificity). Treatment is associated with utility $u\left(h_{h}^{+}\right)$ if the patient's true health state is healthy and with $u\left(h_{d}^{+}\right)$ if they are sick. Without treatment, utility is $u\left(h_{h}^{-}\right)$ if the patient is healthy and $u\left(h_{d}^{-}\right)$ if the patient is sick (Figure 1A). Treatment increases the utility of a sick patient but decreases the utility of a healthy patient because of side effects. We assume the following rank order of utility in the different health states: $u\left(h_{h}^{-}\right)>u\left(h_{h}^{+}\right)>u\left(h_{d}^{+}\right)>u\left(h_{d}^{-}\right)$^3^.

**Figure 2:**
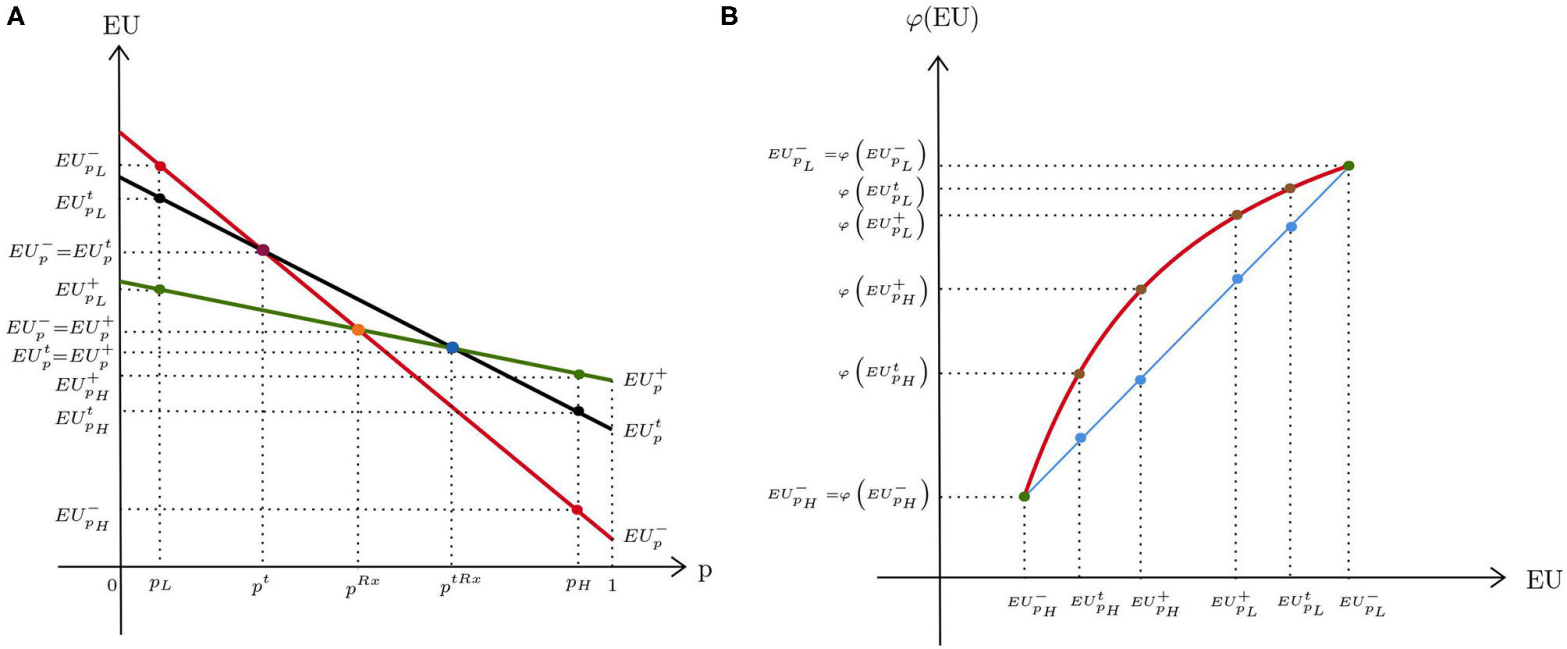
Expected utilities under diagnostic uncertainty: **(A)** ambiguity neutrality; **(B)** ambiguity aversion.

The DM believes that the probability of disease is ambiguous. We assume that the true probability can take two values, *p*_*H*_ and *p*_*L*_, with *p*_*H*_ > *p*_*L*_, which are agreed upon in the medical literature. Based on the information available, the DM assigns beliefs to the probability of disease. μ denotes their subjective probability that *p*_*H*_ is the true probability. 1 − μ is then the belief that the true probability of disease is *p*_*L*_. Let $EU_{p}^{i}$ denote the expected utility for a given probability of disease *p*∈{*p*_*L*_, *p*_*H*_} resulting from a decision *i* ∈ {−, *t*, +}^4^. As illustrated in Figure 2A, our assumptions make sure that whatever decision is taken, the expected utility always decreases in *p*.^5^ We assume that the DM behaves according to the smooth ambiguity model of KMM (9). Compared with an ambiguity-neutral DM, an ambiguity-averse DM will then be more “cautious” with respect to their ex ante evaluations about the true probability of disease. In this framework, let φ be a function defined over expected utilities of decisions reflecting ambiguity attitude^6^. The utilities under no treatment (*V*^−^), diagnostic testing (*V*^*t*^), and treatment (*V*^+^) then become


$$\begin{array}{l} V^{-}=\mu \varphi \left(EU_{p_{H}}^{-}\;\right)+\left(1-\mu \right)\varphi \left(EU_{p_{L}}^{-}\;\right)\\ V^{t}=\mu \varphi \left(EU_{p_{H}}^{t}\;\right)+\left(1-\mu \right)\varphi \left(EU_{p_{L}}^{t}\;\right)\\ V^{+}=\mu \varphi \left(EU_{p_{H}}^{+}\;\right)+\left(1-\mu \right)\varphi \left(EU_{p_{L}}^{+}\;\right). \end{array}$$

With ambiguity aversion, φ is strictly increasing and concave (Figure 2B). The straight line represents an ambiguity-neutral DM for whom φ is linear. For simplicity, φ is scaled such that $\varphi \left(EU_{p}^{-}\;\right)=EU_{p}^{-}$ for *p* ∈ {*p*_*L*_, *p*_*H*_}. Moreover, we assume that at probability *p*_*L*_, both the ambiguity-averse and the ambiguity-neutral DM choose no treatment, whereas at probability *p*_*H*_, both decide to treat without prior testing^7^.

The test has a positive value only if it provides a higher utility than the closest decision alternative chosen in the absence of a test option. For low probabilities, this is no treatment, and for high probabilities, this is treatment. If *V*^*t*^ is compared to *V*^−^ (Figure 2B), the concavity of φ implies that $\varphi \left(EU_{p_{L}}^{-}\;\right)-\;\varphi \left(EU_{p_{L}}^{t}\;\right)$ is smaller than $EU_{p_{L}}^{-}-EU_{p_{L}}^{t}$. That is, ambiguity aversion decreases the advantage of no treatment compared with the test option at low probability of disease *p*_*L*_. When the probability of disease is high, *V*^*t*^ is compared with *V*^+^. In this case, $\varphi \left(EU_{p_{H}}^{+}\;\right)-\;\varphi \left(EU_{p_{H}}^{t}\;\right)$ becomes larger than $EU_{p_{H}}^{+}-EU_{p_{H}}^{t}$, which means that the direct treatment option is more attractive to an ambiguity-averse DM than an ambiguity-neutral DM.

To formally examine the influence of ambiguity aversion on the decision thresholds, let us compare the thresholds for two decision makers, DM1 and DM2, who share the same beliefs (μ and 1−μ) and whose ambiguity attitudes are captured by φ_1_ and φ_2_, respectively. Suppose that the degree of ambiguity aversion of DM2 is larger than that of DM1 (i.e., $-\frac{\varphi_{2}^{\prime \prime }}{\varphi_{2}^{\prime }}>-\frac{\varphi_{1}^{\prime \prime }}{\varphi_{1}^{\prime }}$). The *test thresholds* (referring to the subjective probability of being at high risk) at which the DMs are indifferent between no treatment and testing can then be written as follows:


$$\begin{array}{l} \mu_{1}^{t}=\frac{1}{1+\frac{\varphi_{1}\left(EU_{p_{H}}^{t}\;\right)-\varphi_{1}\left(EU_{p_{H}}^{-}\;\right)}{\varphi_{1}\left(EU_{p_{L}}^{-}\;\right)-\varphi_{1}\left(EU_{p_{L}}^{t}\;\right)}}\\ \mu_{2}^{t}=\frac{1}{1+\frac{\varphi_{2}\left(EU_{p_{H}}^{t}\;\right)-\varphi_{2}\left(EU_{p_{H}}^{-}\;\right)}{\varphi_{2}\left(EU_{p_{L}}^{-}\;\right)-\varphi_{2}\left(EU_{p_{L}}^{t}\;\right)}} \end{array}$$

Following Theorem 1 of Pratt (21), $-\frac{\varphi_{2}^{\prime \prime }}{\varphi_{2}^{\prime }}>-\frac{\varphi_{1}^{\prime \prime }}{\varphi_{1}^{\prime }}$ implies that $\mu_{1}^{t}>\mu_{2}^{t}$. A higher degree of ambiguity aversion thus lowers the subjective probability at which the DM is indifferent between no treatment and diagnostic testing. This result can be explained by the fact that the switch from no treatment to testing reduces the spread of expected utilities, which decreases from $EU_{p_{L}}^{-}-EU_{p_{H}}^{-}$ to $EU_{p_{L}}^{t}-EU_{p_{H}}^{t}$. The more ambiguity averse a DM is, the more that they appreciate this reduction in the spread.

The *test-treatment thresholds* at which DMs are indifferent between prior testing and direct treatment are given by


$$\begin{array}{l} \mu_{1}^{tRx}=\frac{1}{1+\frac{\varphi_{1}\left(EU_{p_{H}}^{+}\;\right)-\varphi_{1}\left(EU_{p_{H}}^{t}\;\right)}{\varphi_{1}\left(EU_{p_{L}}^{t}\;\right)-\varphi_{1}\left(EU_{p_{L}}^{+}\;\right)}}\\ \mu_{2}^{tRx}=\frac{1}{1+\frac{\varphi_{2}\left(EU_{p_{H}}^{+}\;\right)-\varphi_{2}\left(EU_{p_{H}}^{t}\;\right)}{\varphi_{2}\left(EU_{p_{L}}^{t}\;\right)-\varphi_{2}\left(EU_{p_{L}}^{+}\;\right)}}. \end{array}$$

As long as $-\frac{\varphi_{2}^{\prime \prime }}{\varphi_{2}^{\prime }}>-\frac{\varphi_{1}^{\prime \prime }}{\varphi_{1}^{\prime }}$, Theorem 1 of Pratt (21) ensures that $\mu_{1}^{tRx}>\mu_{2}^{tRx}$. In other words, the test-treatment threshold decreases in the degree of ambiguity aversion. In the transition from testing to direct treatment, the spread in expected utilities decreases because $EU_{p_{L}}^{+}-EU_{p_{H}}^{+}$ is smaller than $EU_{p_{L}}^{t}-EU_{p_{H}}^{t}$, which increases the propensity of an ambiguity-averse DM for direct treatment. A proof of the more general case with more than two values for the probability of disease is presented in Appendix A.1.

## Decision Thresholds Under Therapeutic Ambiguity

In the previous analysis, we assumed that the treatment is always successful. In medical practice, however, the treatment outcome is often uncertain because complications may arise with invasive treatment in particular. Sometimes, there are diagnostic tests that allow the DM to infer the probability of a successful treatment. For instance, pulmonary function tests are performed before surgery in order to assess surgical risk or the likelihood of post-operative complications^8^.

**Figure 3:**
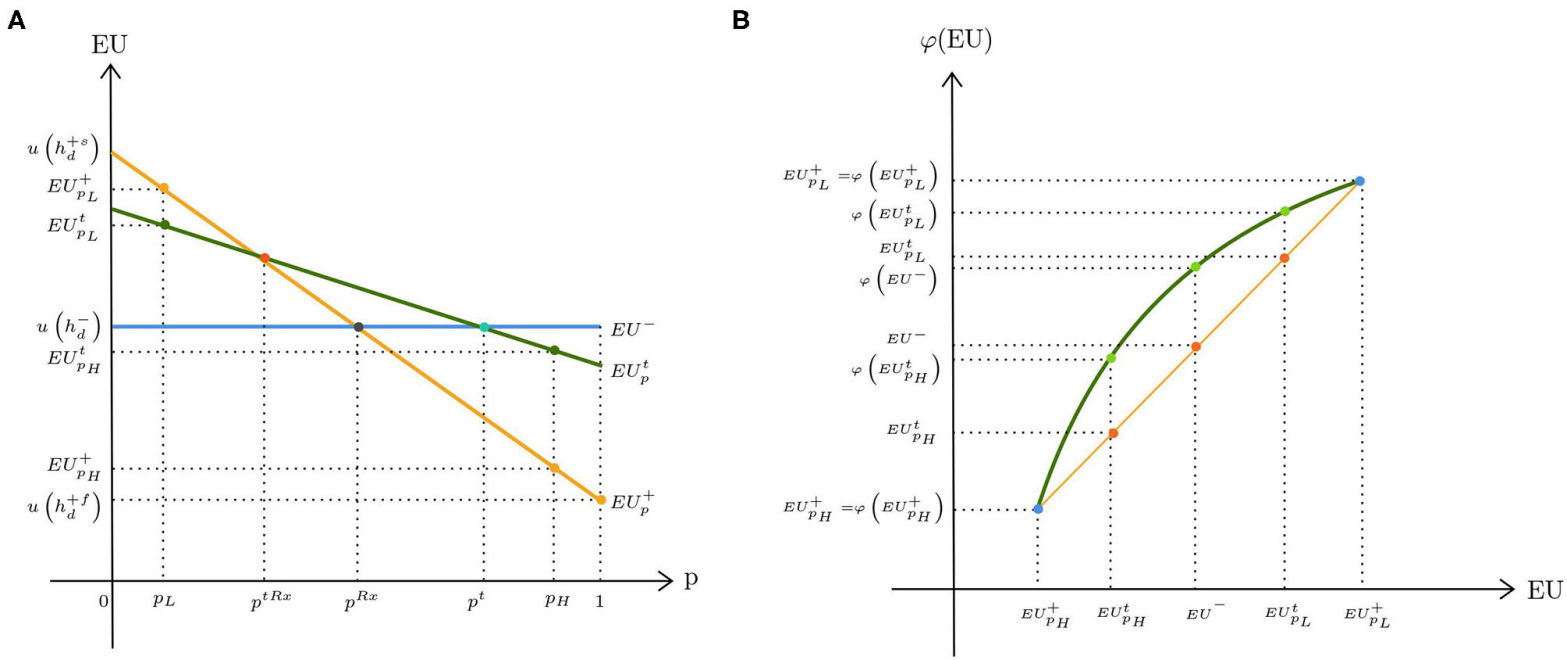
Expected utilities under therapeutic uncertainty: **(A)** ambiguity neutrality; **(B)** ambiguity aversion.

The standard model with therapeutic risk as introduced by Eeckhoudt (16) assumes that the patient's ex ante health status is diseased with certainty and that the corresponding utility is $u\left(h_{d}^{-}\right)$. The only source of uncertainty is the outcome of treatment that is due to the therapeutic hazard: if treatment fails (*f* ), patients may face a serious health deterioration or even death. If treatment is successful (*s*), health improves. Let $u\left(h_{d}^{+s}\right)$ and $u\left(h_{d}^{+f}\right)$ denote the utilities resulting from successful and failed treatment, respectively. We assume that $u\left(h_{d}^{+s}\right)>u\left(h_{d}^{-}\right)>u\left(h_{d}^{+f}\right)$ holds. Moreover, there is a medical test that correctly detects a failed outcome of treatment with probability *Se* (sensitivity) and successful treatment with probability *Sp* (specificity)^9^. The DM decides on the treatment in accordance with the test outcome (treatment if negative and no treatment if positive)^10^. The decision tree for the test is presented in Figure 1B. The true probability of treatment failure is unknown because of missing or conflicting medical data. However, the DM believes that there are two possible values for the failure rate, *p*_*H*_ and *p*_*L*_, with *p*_*H*_ > *p*_*L*_. Following the framework of the previous section, suppose that μ and 1−μ denote the subjective belief of the DM that *p*_*H*_ and *p*_*L*_ are the true probabilities of treatment failure, respectively. Figure 3A illustrates the expected utilities resulting from the decisions of no treatment, *EU*^−^, testing, $EU_{p}^{t}$, and treatment, $EU_{p}^{+}$ as a function of the failure rate *p*^11^. The horizontal line corresponds to the expected utility of no treatment, which does not depend on *p*. Both expected utilities of treatment $\left(EU_{p}^{+}\right)$ and testing $EU_{p}^{t}$ decrease with *p*. Furthermore, it can be shown that $\left(EU_{p}^{+}\right)$is steeper than $EU_{p}^{t}$, which indicates that testing reduces the spread in expected utilities compared with direct treatment. According to the smooth ambiguity model, the resulting utilities can be written as follows:


$$\begin{array}{l} V^{-}=\varphi \left(EU^{-}\right)=\varphi \left(u\left(h_{d}^{-}\right)\right)\\ V^{t}=\mu \varphi \left(EU_{p_{H}}^{t}\;\right)+\left(1-\mu \right)\varphi \left(EU_{p_{L}}^{t}\;\right)\\ V^{+}=\mu \varphi \left(EU_{p_{H}}^{+}\;\right)+\left(1-\mu \right)\varphi \left(EU_{p_{L}}^{+}\;\right). \end{array}$$

For low probabilities of treatment failure, treatment is the dominant strategy. Hence, *V*^*t*^ is compared with *V*^+^. When the failure rate is high, *V*^*t*^ is compared with *V*^−^. Figure 3B depicts the change in the resulting utilities under ambiguity aversion. The straight line corresponds to the case of ambiguity neutrality (i.e., φ is linear). We scaled φ such that $\varphi \left(EU_{p}^{+}\right)=EU_{p}^{+}$ for *p* ∈ {*p*_*L*_, *p*_*H*_}. At the lower range of the failure rate, ambiguity aversion makes direct treatment less attractive than prior testing. This can be inferred from the figure because $\varphi \left(EU_{p_{L}}^{+}\right)-\varphi \left(EU_{p_{L}}^{t}\right)$ is smaller than $EU_{p_{L}}^{+}-EU_{p_{L}}^{t}$. On the other hand, at the higher range of the failure rate, $\varphi \left(EU^{-}\right)-\varphi \left(EU_{p_{H}}^{t}\right)$ exceeds $EU^{-}-EU_{p_{H}}^{t}$, which implies that the utility gain of no treatment is more pronounced for an ambiguity-averse DM when the therapeutic ambiguity is high.

More formally, we can define the decision thresholds when there is a therapeutic ambiguity as follows: The *test threshold* corresponds to the subjective probability of being at high risk (μ) at which *V*^*t*^ = *V*^−^. When μ is above this threshold, no treatment is the preferred strategy because treatment is highly risky. Similarly, the *test-treatment threshold* refers to the subjective probability that the true failure rate is *p*_*H*_ at which *V*^+^ = *V*^*t*^. Above this threshold, the DM prefers testing before deciding on the treatment. Suppose that the degree of ambiguity aversion of DM2 exceeds that of DM1, i.e., $-\frac{\varphi_{2}^{\prime \prime }}{\varphi_{2}^{\prime }}>-\frac{\varphi_{1}^{\prime \prime }}{\varphi_{1}^{\prime }}$. We can write their test thresholds as follows:


$$\begin{array}{l} \mu_{1}^{t}=\frac{1}{1+\frac{\varphi_{1}\left(EU^{-}\;\right)-\varphi_{1}\left(EU_{p_{H}}^{t}\;\right)}{\varphi_{1}\left(EU_{p_{L}}^{t}\;\right)-\varphi_{1}\left(EU^{-}\;\right)}}\\ \mu_{2}^{t}=\frac{1}{1+\frac{\varphi_{2}\left(EU^{-}\;\right)-\varphi_{2}\left(EU_{p_{H}}^{t}\;\right)}{\varphi_{2}\left(EU_{p_{L}}^{t}\;\right)-\varphi_{2}\left(EU^{-}\;\right)}} \end{array}$$

φ_2_ being more concave than φ_1_ ensures that $\mu_{1}^{t}>\mu_{2}^{t}$. Hence, ambiguity aversion makes no treatment more appealing than testing. When no treatment is the preferred strategy, the resulting utility is $u\left(h_{d}^{-}\right)$ with certainty. The more ambiguity averse the DM is, the more that they value this certainty.

Similarly, the test-treatment thresholds of the two DMs become


$$\begin{array}{l} \mu_{1}^{tRx}=\frac{1}{1+\frac{\varphi_{1}\left(EU_{p_{H}}^{t}\;\right)-\varphi_{1}\left(EU_{p_{H}}^{+}\;\right)}{\varphi_{1}\left(EU_{p_{L}}^{+}\;\right)-\varphi_{1}\left(EU_{p_{L}}^{t}\;\right)}}\\ \mu_{2}^{tRx}=\frac{1}{1+\frac{\varphi_{2}\left(EU_{p_{H}}^{t}\;\right)-\varphi_{2}\left(EU_{p_{H}}^{+}\;\right)}{\varphi_{2}\left(EU_{p_{L}}^{+}\;\right)-\varphi_{2}\left(EU_{p_{L}}^{t}\;\right)}}. \end{array}$$

As before, we can show that $\mu_{1}^{tRx}>\mu_{2}^{tRx}$. That is, DM2 is less tolerant to the chance of treatment failure so that they switch from direct treatment to prior testing at a lower subjective belief of it being high risk. It can also be shown that in the presence of therapeutic risk, the spread of expected utilities is smaller under testing than under treatment, which makes an ambiguity-averse DM more inclined to test. A proof of the more general case with more than two values for the failure rate is presented in Appendix A.2.

## Discussion

Although medical decisions are often made under ambiguity about the true probability distribution of outcomes, there has been a lack of theoretical model in the literature that analyzes the effect of such ambiguity on the test and treatment decisions. This paper intends to close this gap by presenting a model of medical decision based on the KMM approach to ambiguity (13). We chose the KMM model because it distinguishes between ambiguity and ambiguity attitudes and allows for clear-cut comparative statics. Several alternative non-expected utility models have been developed to accommodate ambiguity aversion. Examples include maxmin expected utility model (27), α−maxmin expected utility model (28), and prospect theory (29)^12^. They all carry the basic intuition that ambiguity aversion reinforces the pessimism about the probability of disease and the probability of treatment failure. They should therefore share the result that an ambiguity-averse DM is more prone to treatment when there is diagnostic ambiguity and vice versa under therapeutic ambiguity^13^. The effect of ambiguity aversion on the propensity to test is less clear. The (subjective) probability interval for which DMs opt for the test shifts under both diagnostic and therapeutic ambiguity. However, it is unclear whether these intervals shrink or widen; the effect on the demand for the test cannot be signed.

Our analysis concentrates on the effect of ambiguity aversion on decision thresholds for a given level of ambiguity. Another issue is the effect of an increase in the ambiguity, i.e., a mean-preserving spread of *p*_*L*_ and *p*_*H*_, for a constant degree of ambiguity aversion. In this case, the spread of expected utilities would increase the most for no treatment (treatment) under diagnostic uncertainty (therapeutic uncertainty), reinforcing our results on the thresholds. Thus, the magnitude of changes in the thresholds increases in the level of ambiguity.

The specific source of ambiguity with regard to the acquisition of new information also plays a role for the demand of a diagnostic test. Nocetti (31) distinguishes between conditional ambiguity over the true probabilities of outcomes and unconditional ambiguity attached to the test outcomes. In the main text, we presented conditional ambiguity models in which the only source of ambiguity is the uncertainty over the correct probability distribution that remains after the acquisition of imperfect test outcomes. Hoy et al. (20) model ambiguity such that the DM is indifferent to the spread in expected utilities caused by the multiplicity of possible probabilities of disease but is averse to the spread resulting from ex ante uncertain test results (i.e., unconditional ambiguity aversion). They cannot sign the effect of ambiguity aversion on the value of new information. We show in Appendices A.3, A.4 that unconditional ambiguity aversion decreases the demand for testing both under diagnostic and therapeutic ambiguity^14^. This is because an unconditional ambiguity-averse DM dislikes the spread resulting from updating prior beliefs according to the test outcome. Instead of “living through” this ambiguity by taking the test, they prefer standing by their pre-existing beliefs. As a result, they are inclined to choose the default action (e.g., no treatment as opposed to testing under low prior probabilities of disease). These findings are in line with Golman et al. (32), who claim that people may avoid medical tests that provide free information if they fear the possible stress and anxiety attached to the results. However, the concept of unconditional ambiguity is not fully compatible with the KMM framework and requires an extension^15^. Furthermore, the experimental irregularity documented as ambiguity aversion in the literature refers to the situations in which the DM dislikes the uncertainty of the correct probability distribution of final outcomes, which we dealt with in the main text. The empirical relevance of different notions of ambiguity attitude also depends on the context. Fels (33) shows that the availability of an effective treatment substantially reduces the information avoidance and increases the uptake rate of the test. Because in our models, there is a treatment option that improves health in the sick state, ignoring the impact of negative emotions related to the uncertainty of test results on decision-making appears to be a reasonable assumption.

The scope of our results depends on whether ambiguity aversion is regarded to be irrational. Some argue that ambiguity aversion results from the behavioral bias leading to information avoidance and thus a departure from rationality (30, 34). The opponents to this view assert that, acknowledging the obscurity of true probabilities in some circumstances, a DM is right to be cautious about their somewhat arbitrarily chosen beliefs (35). Notwithstanding, ambiguity aversion leads to welfare losses (17). Fleurbaey (36) argues that ambiguity aversion is not admissible in the context of social decision-making that concerns the admission and reimbursement of medical interventions or the establishment of medical guidelines. Public regulators should not be allowed to let their anxiety about uncertainty affect their recommendations and decisions, although physicians in private practice may deviate from the expected utility theory when deciding on the treatment strategy if they act on behalf of their ambiguity-averse patient as long as only the doctor–patient dyad is at stake. However, if the corresponding decisions have financial consequences for statutory health insurance, ambiguity aversion is costly and needs to be ignored by medical providers in order to minimize inefficiencies.

## Conclusion

Attitudes toward ambiguity become significantly relevant in medical decision-making whenever agents have to deal with uncertainty over the correct probability distribution of outcomes. In this regard, a medical test can be seen as an information service that may reduce or even completely resolve ambiguity. In the present paper, using the smooth ambiguity model of KMM (13), we analyzed how decision makers react to such information and how this reaction changes with the degree of ambiguity aversion.

For diagnostic ambiguity aversion, we have shown that it makes diagnostic testing more attractive when the default option is not to treat. This is because the spread of expected utilities is lower under diagnostic testing than under no treatment. This is more appreciated by an ambiguity-averse DM. However, when the default option is treatment, prior testing becomes less favorable for an ambiguity-averse DM because it increases the spread in expected utilities compared to direct treatment. With therapeutic ambiguity aversion, the DM is more prone to not treat and abstain from testing when the default option is not to treat and less prone to direct treatment when the default option is treatment. Intuitively, ambiguity aversion reduces the tolerance toward treatment failure.

An important extension for future research would be to relax the assumption of a universal ambiguity attitude. The joint occurrence of ambiguity seeking for unlikely events and ambiguity aversion for more likely events has been observed (37, 38). It is unclear as to whether our results would prevail. Further evidence is needed to determine which ambiguity attitude is more relevant in different clinical settings.

## Data Availability Statement

The original contributions presented in the study are included in the article/Supplementary Material, further inquiries can be directed to the corresponding author.

## Author Contributions

The authors have collaborated on every stage, discussing the idea, the scope of the contribution, and writing the paper. Both authors contributed to the article and approved the submitted version.

## Conflict of Interest

The authors declare that the research was conducted in the absence of any commercial or financial relationships that could be construed as a potential conflict of interest.

## Publisher's Note

All claims expressed in this article are solely those of the authors and do not necessarily represent those of their affiliated organizations, or those of the publisher, the editors and the reviewers. Any product that may be evaluated in this article, or claim that may be made by its manufacturer, is not guaranteed or endorsed by the publisher.

## Footnotes

^1^Additionally, Attema et al. (10) provided experimental evidence that ambiguity aversion is more pronounced for health losses than for health gains. Baillon et al. (11) and Courbage and Peter (12) studied the effect of ambiguity aversion on prevention decisions for reducing health risks.

^2^Fujii and Osaki (18) confirmed this observation in an extended model with comorbidity risk between a primary disease and secondary diseases.

^3^A more general assumption, $u\left(h_{h}^{-}\right)>u\left(h_{h}^{+}\right)$ and $u\left(h_{d}^{+}\right)>u\left(h_{d}^{-}\right)$, without imposing a sorting between health states resulting from treatment would not change our result.

^4^In explicit terms: $EU_{p_{j}}^{+}=p_{j}u\left(h_{d}^{+}\right)+\left(1-p_{j}\right)u\left(h_{h}^{+}\right)$, $EU_{p_{j}}^{-}=p_{j}u\left(h_{d}^{-}\right)+\left(1-p_{j}\right)u\left(h_{h}^{-}\right)$, and $EU_{p_{j}}^{t}=p_{j}\left[Se\;u\left(h_{d}^{+}\right)+\left(1-Se\right)u\left(h_{d}^{-}\right)\right]+\left(1-p_{j}\right)\left[Sp\;u\left(h_{h}^{-}\right)+\left(1-Sp\right)u\left(h_{h}^{+}\right)\right]$, where *j* ∈ {*H, L*}.

^5^Furthermore, $\left|\frac{\partial EU^{+}}{\partial p}|<|\frac{\partial EU^{t}}{\partial p}|<|\frac{\partial EU^{-}}{\partial p}\right|$ holds; i.e., $EU_{p}^{-}$ is the steepest line followed by $EU_{p}^{t}$ and $EU_{p}^{+}$.

^6^The model by KMM conveniently separates ambiguity (captured by the subjective beliefs of the DM) and ambiguity attitude (characterized by φ), which in turn allows a comparison of two DMs with different ambiguity attitudes.

^7^This assumption, although convenient for illustration purposes, is not necessary for the results of the paper as we show in Appendix A.1.

^8^See Powell and Caplan (22), Bolliger (23), Zhang et al. (24), Bapoje et al. (25), and Myers et al. (26).

^9^Because the test identifies patients with existing medical conditions that put them at risk for treatment complications, associating the sensitivity with treatment failure seems to be a reasonable assumption.

^10^This assumption can be relaxed so that instead of no treatment, less invasive treatment options can be applied in the case of a positive test result. However, this alternative treatment would not be as risky and as effective as the original treatment option.

^11^Explicitly, $EU_{p_{j}}^{-}=u\left(h_{d}^{-}\right)$, $EU_{p_{j}}^{t}=p_{j}\left[Se\;u\left(h_{d}^{-}\right)+\left(1-Se\right)u\left(h_{d}^{+f}\;\right)\right]+\left(1-p_{j}\right)\;\left[Sp\;u\;\left(h_{d}^{+s}\;\right)+\left(1-Sp\right)u\left(h_{d}^{-}\right)\;\right]$, and $EU_{p_{j}}^{+}=p_{j}u\left(h_{d}^{+f}\right)+\left(1-p_{j}\right)u\left(h_{d}^{+s}\right)$, where *j* ∈ {*H, L*}.

^12^The descriptive nature of non-expected utility models has also been questioned and criticized for creating more paradoxes than they intend to resolve. See Al-Najjar and Weinstein (30) for an extensive review of this matter.

^13^Our conclusions hold under maxmin expected utility model as it is a special case of KMM model with infinite ambiguity. The α-maxmin model shares the pessimistic interpretation, although it does not allow for comparative statistics with EUT. Berger et al. (17) showed in the Appendix that the sign of change in the treatment threshold is the same under the KMM model and prospect theory, which also applies to our model.

^14^Hoy et al. (20) did not derive the test and test-treatment thresholds, which explain why they did not determine the demand for testing and its change due to ambiguity aversion.

^15^Nocetti (31) presents a model that accommodates both types of ambiguity aversion. However, he also emphasizes the necessary deviation from the KMM model in order to incorporate unconditional ambiguity aversion, because a DM with the preferences of KMM is always unconditional ambiguity neutral.

## References

[1] Knight FH. Risk, Uncertainty and Profit. Boston; New York, NY: Houghton Mifflin (1921). p. 406.

[2] Ellsberg D. Risk, ambiguity, and the savage axioms. Q J Econ. (1961) 75:643–69. 10.2307/1884324

[3] Curley SP Eraker SA Yates JF. An investigation of patient's reactions to therapeutic uncertainty. Med Decis Making. (1984) 4:501–11. 10.1177/0272989X8400400412

[4] Curley SP Young MJ Yates JF. Characterizing physicians' perceptions of ambiguity. Med Decis Making. (1989) 9:116–24. 10.1177/0272989X8900900206 2747448

[5] Ritov I Baron J. Reluctance to vaccinate: omission bias and ambiguity. J Behav Decis Making. (1990) 3:263–77. 10.1002/bdm.3960030404

[6] Viscusi WK Magat WA Huber J. Communication of ambiguous risk information. Theory Decis. (1991) 31:159–73. 10.1007/BF00132991

[7] Viscusi WK Magat WA. Bayesian decisions with ambiguous belief aversion. J Risk Uncertain. (1992) 5:371–87. 10.1007/BF00122576

[8] Gerrity MS DeVellis RF Earp JA. Physicians' reactions to uncertainty in patient care: a new measure and new insights. Med Care. (1990) 28:724–36. 10.1097/00005650-199008000-00005 2385142

[9] Portnoy DB Han PKJ Ferrer RA Klein WMP Clauser SB. Physicians' attitudes about communicating and managing scientific uncertainty differ by perceived ambiguity aversion of their patients. Health Expect. (2013) 16:362–72. 10.1111/j.1369-7625.2011.00717.x 21838835 PMC3218256

[10] Attema AE Bleichrodt H L'Haridon O. Ambiguity preferences for health. Health Econ. (2018) 27:1699–716. 10.1002/hec.3795 29971896 PMC6221042

[11] Baillon A Bleichrodt H Emirmahmutoglu A Jaspersen J Peter R. When risk perception gets in the way: probability weighting and underprevention. Operat Res. (2020) 1–22. 10.1287/opre.2019.1910

[12] Courbage C Peter R. On the effect of uncertainty on personal vaccination decisions. Health Econ. (2021) 30:2937–42. 10.2139/ssrn.3821166 34346125 PMC9290645

[13] Klibanoff P Marinacci M Mukerji S. A smooth model of decision making under ambiguity. Econometrica. (2005) 73:1849–92. 10.1111/j.1468-0262.2005.00640.x31136854

[14] Neilson WS. Ambiguity Aversion: An Axiomatic Approach Using Second Order Probabilities. Knoxville: Mimeo (1993).

[15] Pauker SG Kassirer JP. The threshold approach to clinical decision making. N Engl J Med. (1980) 302:1109–17. 10.1056/NEJM198005153022003 7366635

[16] Eeckhoudt L. Risk and Medical Decision Making. Boston, MA: Springer US (2002).

[17] Berger L Bleichrodt H Eeckhoudt L. Treatment decisions under ambiguity. J Health Econ. (2013) 32:559–69. 10.1016/j.jhealeco.2013.02.001 23537710

[18] Fujii Y Osaki Y. The willingness to pay for health improvement under comorbidity ambiguity. J Health Econ. (2019) 66:91–100. 10.1016/j.jhealeco.2019.04.002 31136854

[19] Snow A. Ambiguity and the value of information. J Risk Uncertain. (2010) 40:133–45. 10.1007/s11166-010-9088-7

[20] Hoy M Peter R Richter A. Take-up for genetic tests and ambiguity. J Risk Uncertain. (2014) 48:111–33. 10.1007/s11166-014-9186-z

[21] Pratt JW. Risk aversion in the small and in the large. Econometrica. (1964) 32:122–36. 10.2307/1913738

[22] Caplan CE. Pulmonary function tests in preoperative pulmonary evaluation. Clin Chest Med. (2001) 22:703–14. 10.1016/S0272-5231(05)70061-7 11787660

[23] Bolliger CT. Evaluation of operability before lung resection. Curr Opin Pulm Med. (2003) 9:321–6. 10.1097/00063198-200307000-00013 12806247

[24] Zhang JG Wang W Qiu GX Wang YP Weng XS Xu HG. The role of preoperative pulmonary function tests in the surgical treatment of scoliosis. Spine. (2005) 30:218–21. 10.1097/01.brs.0000150486.60895.a1 15644760

[25] Bapoje SR Whitaker JF Schulz T Chu ES Albert RK. Preoperative evaluation of the patient with pulmonary disease. Chest. (2007) 132:1637–45. 10.1378/chest.07-0347 17998364

[26] Myers PO Tissot C Beghetti M. Assessment of operability of patients with pulmonary arterial hypertension associated with congenital heart disease: do we have the good tools to predict success? Circ J. (2014) 78:4–11. 10.1253/circj.CJ-13-1263 24225339

[27] Gilboa I Schmeidler D. Maxmin expected utility with non-unique prior. J Math Econ. (1989) 18:141–53. 10.1016/0304-4068(89)90018-9

[28] Ghirardato P Maccheroni F Marinacci M. Differentiating ambiguity and ambiguity attitude. J Econ Theory. (2004) 118:133–73. 10.1016/j.jet.2003.12.004

[29] Tversky A Kahneman D. Advances in prospect theory: cumulative representation of uncertainty. J Risk Uncertain. (1992) 5:297–323. 10.1007/BF0012257415795132

[30] Al-Najjar NI Weinstein J. The ambiguity aversion literature: a critical assessment. Econ Philos. (2009) 25:249–84. 10.1017/S026626710999023X

[31] Nocetti DC. Ambiguity and the value of information revisited. Geneva Risk Ins Rev. (2018) 43:25–38. 10.1057/s10713-018-0025-z

[32] Golman R Hagmann D Loewenstein G. Information avoidance. J Econ Lit. (2017) 55:96–135. 10.1257/jel.20151245

[33] Fels M. On the value of information: why people reject medical tests. J Behav Exp Econ. (2015) 56:1–12. 10.1016/j.socec.2015.02.006

[34] Wakker PP. Prospect Theory: For Risk and Ambiguity. Cambridge: Cambridge University Press (2010).

[35] Gilboa I Marinacci M. Ambiguity and the Bayesian paradigm. In: Arló-Costa H Hendricks VF van Benthem J, editors. Readings in Formal Epistemology: Sourcebook. Cham: Springer International Publishing (2016). p. 385–439.

[36] Fleurbaey M. Welfare economics, risk and uncertainty. Can J Economics. (2018) 51:5–40. 10.1111/caje.12314

[37] Kocher MG Lahno AM Trautmann ST. Ambiguity aversion is not universal. Eur Econ Rev. (2018) 101:268–83. 10.1016/j.euroecorev.2017.09.016

[38] Trautmann ST van de Kuilen G. Ambiguity attitudes. In: Keren G Wu G, editors. The Wiley Blackwell Handbook of Judgment and Decision Making. Chichester: John Wiley & Sons, Ltd. (2015). p. 89–116

