# Supplementary material for: Decision Thresholds for Medical Tests Under Ambiguity Aversion
Source: Front Health Serv. 2022 Mar 21;2:825315. doi: 10.3389/frhs.2022.825315 (PMC10012708; doi:10.3389/frhs.2022.825315)
Supplement: Supplementary file 1 [file Data_Sheet_1.PDF]

Appendix

A Formal Proofs

A.1 Diagnostic Ambiguity

Below, we provide a formal proof of the result in the presence of diagnostic uncertainty. Compared with ambiguity neutrality, ambiguity aversion decreases both the test and the test-treatment thresholds.

Suppose that there are *n* possible probabilities of disease with $p_1 < \ldots < p_\mathrm{n}$. Let μ denote the subjective probability distribution (beliefs) of an ambiguity averse DM over the probabilities of disease. $\mathrm{supp}(\mu)$ then denotes the set of probabilities for which the DM assigns a positive value (i.e., the probabilities p_i for which $\mu(p_i) > 0$). Let φ be a concave function defined over expected utilities defining ambiguity aversion. The utilities under treatment, diagnostic testing and no treatment can then be written as follows:

$$V^+ = \sum_{supp(\mu)} \mu(p_i)\varphi\left(EU_{p_i}^+\right)$$
$$V^t = \sum_{supp(\mu)} \mu(p_i)\varphi\left(EU_{p_i}^t\right)$$
$$V^- = \sum_{supp(\mu)} \mu(p_i)\varphi\left(EU_{p_i}^-\right)$$

The DM decides to test as opposed to not treating whenever $V^t \geq V^-$ and decides to treat without prior testing whenever $V^+ \geq V^t$.

Claim 1. Test threshold under ambiguity aversion is lower than test threshold under ambiguity neutrality. Assuming that both decision makers share the same beliefs, this means if DM2 is more ambiguity averse than DM1, DM2 is more inclined to test than DM1 such that

(i) if DM1 decides to test as opposed to no treatment, DM2 will also do so
(ii) if DM1 is indifferent between testing and no treatment, DM2 will decide to test
(iii) there is a (subjective) probability of disease at which DM1 decides not to treat without prior testing and DM2 decides to test

Proof of Claim 1. It can easily be shown that the difference $\varphi\left(EU_p^t\right) - \varphi\left(EU_p^-\right)$ is negative for $p = 0$, positive for $p = 1$, and increases with p. Together with the continuity of *u* and φ, this implies that $\exists\, p^*, \varphi\left(EU_{p^*}^t\right) = \varphi\left(EU_{p^*}^-\right) = \hat{\varphi}$. Let *g* be an increasing and concave function. By the mean value theorem, $\forall p \in supp(\mu), \exists c$ between $\varphi\left(EU_p^t\right)$ and $\varphi\left(EU_p^-\right)$ such that $g\left(\varphi\left(EU_p^t\right)\right) - g\left(\varphi\left(EU_p^-\right)\right) \geq g'(c)\left(\varphi\left(EU_p^t\right) - \varphi\left(EU_p^-\right)\right)$. For $p < p^*$, $\hat{\varphi} < \varphi\left(EU_p^t\right) < c < \varphi\left(EU_p^-\right)$. By the concavity of *g*, this implies $g'(\hat{\varphi}) \geq g'(c)$. Hence, $g\left(\varphi\left(EU_p^t\right)\right) - g\left(\varphi\left(EU_p^-\right)\right) \geq g'(\hat{\varphi})\left(\varphi\left(EU_p^t\right) - \varphi\left(EU_p^-\right)\right)$. For

$p > p^*$, $\hat{\varphi} > \varphi(EU_p^t) > c > \varphi(EU_p^-)$. By concavity of g this implies $g'(\hat{\varphi}) \leq g'(\hat{c})$. Hence, $g\left(\varphi(EU_p^t)\right) - g\left(\varphi(EU_p^-)\right) \geq g'(\hat{\varphi})\left(\varphi(EU_p^t) - \varphi(EU_p^-)\right)$. It follows that for all p,

$$\sum_{supp(\mu)} \mu(p_i)[g\left(\varphi(EU_{p_i}^t)\right) - g\left(\varphi(EU_{p_i}^-)\right)] \geq g'(\hat{\varphi}) \sum_{supp(\mu)} \mu(p_i)[\varphi(EU_{p_i}^t) - \varphi(EU_{p_i}^-)]$$

Let V_1^j denote the utility of DM1 and V_2^j denote the utility of DM2 under the decision j. The above expression then becomes $V_2^t - V_2^- \geq g'(\hat{\varphi})(V_1^t - V_1^-)$. Because g is increasing, if $V_1^t - V_1^- > 0$, $V_2^t - V_2^- > 0$. In other words, whenever DM1, whose ambiguity aversion is captured by φ, chooses to test, the more ambiguity-averse DM2, whose ambiguity aversion is captured by an increasing and concave transformation of φ, will also choose to test. Together with the continuity of the utility function, this concludes the proof.

Claim 2. The test-treatment threshold under ambiguity aversion is lower than the test-treatment threshold under ambiguity neutrality. Assuming that both decision makers share the same beliefs, this means that if DM2 is more ambiguity averse than DM1, DM2 is more inclined to treat without prior testing compared to DM1 such that

(i) if DM1 chooses the direct treatment as opposed to prior testing, DM2 will also do so
(ii) if DM1 is indifferent between direct treatment and testing, DM2 will decide to treat
(iii) there is a (subjective) probability of disease at which DM1 decides to test while DM2 chooses to treat directly

Proof of Claim 2. $\varphi(EU_p^+) - \varphi(EU_p^t)$ is negative for $p = 0$, positive for $p = 1$ and increases in p. Together with continuity of u and φ, this implies that $\exists p^*, \varphi(EU_{p^*}^+) = \varphi(EU_{p^*}^t) = \hat{\varphi}$. Let g be an increasing and concave function. By the mean value theorem, $\forall p \in supp(\mu)$, $\exists c$ between $\varphi(EU_p^+)$ and $\varphi(EU_p^t)$ such that $g\left(\varphi(EU_p^+)\right) - g\left(\varphi(EU_p^t)\right) = g'(c)\left(\varphi\left(EU_p^+\right) - \varphi(EU_p^t)\right)$. For $< p^*$, $\hat{\varphi} < \varphi(EU_p^+) < c < \varphi(EU_p^t)$. By concavity of g this implies $g'(\hat{\varphi}) \geq g'(c)$. Hence, $g\left(\varphi(EU_p^+)\right) - g(\varphi(EU_p^t)) \geq g'(\hat{\varphi})(\varphi(EU_p^+) - \varphi(EU_p^t))$. For $p > p^*$, $\hat{\varphi} > \varphi(\mathrm{EU_p^+}) > \mathrm{c} > \varphi(\mathrm{EU_p^t})$. By the concavity of g this implies $g'(\hat{\varphi}) \leq g'(c)$. Hence, $g(\varphi(EU_p^+)) - g(\varphi(EU_p^t)) \geq g'(\hat{\varphi})(\varphi(EU_p^+) - \varphi(EU_p^t))$. It follows that

$$\sum_{supp(\mu)} \mu(p_i)[g\left(\varphi(EU_{p_i}^+)\right) - g\left(\varphi(EU_{p_i}^t)\right)] \geq g'(\hat{\varphi}) \sum_{supp(\mu)} \mu(p_i)[\varphi(EU_{p_i}^+) - \varphi(EU_{p_i}^t)]$$

Let V_1^j denote the utility of DM1 and V_2^j denote the utility of DM2 under the treatment decision j. The above expression then becomes $V_2^+ - V_2^t \geq g'(\hat{\varphi})(V_1^+ - V_1^t)$. Because g is increasing, if $V_1^+ - V_1^t > 0$, $V_2^+ - V_2^t > 0$. In other words, whenever DM1, whose ambiguity aversion is captured by φ, chooses direct treatment, the more ambiguity-averse DM2, whose ambiguity aversion is captured by an increasing and concave transformation of φ, will also choose to treat. Together with the continuity of the utility function, this concludes the proof.

A.2 Therapeutic Ambiguity

Below, we provide a formal proof of the result in the presence of therapeutic uncertainty. Compared with ambiguity neutrality, ambiguity aversion increases both the test and the test-treatment threshold.

Suppose that there is a therapeutic ambiguity and there are *n* possible probabilities of treatment failure with $p_1 < \ldots < p_n$. Let μ denote the subjective probability distribution (beliefs) of an ambiguity averse DM over the failure rates and φ be a concave function defined over expected utilities denoting ambiguity aversion. The utilities under treatment, testing (which gives information about a possible treatment failure), and no treatment then become

$$V^+ = \sum_{supp(\mu)} \mu(p_i)\varphi\left(EU_{p_i}^+\right)$$
$$V^t = \sum_{supp(\mu)} \mu(p_i)\varphi\left(EU_{p_i}^t\right)$$
$$V^- = \varphi\left(u(h_d^-)\right)$$

The DM decides to test first as opposed to no treatment whenever $V^t \geq V^-$ and decides to treat without prior testing whenever $V^+ \geq V^t$.

Claim 3. The test threshold (referring to the failure rate) under therapeutic ambiguity aversion is lower than the test threshold under ambiguity neutrality. Assuming that both decision makers share the same beliefs, this means that if DM2 is more ambiguity-averse than DM1, DM2 is less inclined to test than DM1 such that

(i) if DM1 decides not to treat without prior testing, DM2 will also do so
(ii) if DM1 is indifferent between testing and no treatment, DM2 will choose no treatment
(iii) there is a (subjective) probability of failure at which DM1 decides to test while DM2 chooses no treatment

Proof of Claim 3. $\varphi(EU^-) - \varphi\left(EU_p^t\right)$ is negative for $p = 0$, positive for $p = 1$ and increases in p. Together with continuity of u and φ, this implies that $\exists p^*, \varphi\left(EU_{p^*}^t\right) = \varphi(u(h_d^-))$. Let g be an increasing and concave function. By the mean value theorem, $\forall p \in supp(\mu), \exists c$ between $\varphi\left(EU_p^+\right)$ and $\varphi(u(h_d^-))$ such that $g(\varphi(u(h_d^-))) - g\left(\varphi\left(EU_p^t\right)\right) = g'(c)\left(\varphi(u(h_d^-)) - \varphi\left(EU_p^t\right)\right)$. For $< p^*$, $\varphi\left(EU_p^t\right) > c > \varphi(u(h_d^-))$. By concavity of g this implies $g'(\varphi(u(h_d^-))) \geq g'(c)$. Hence, $g\left(\varphi\left(u(h_d^-)\right)\right) - g\left(\varphi\left(EU_p^t\right)\right) \geq g'(\varphi(u(h_d^-)))\left(\varphi(u(h_d^-)) - \varphi\left(EU_p^t\right)\right)$. For $p > p^*$, $\varphi\left(EU_p^t\right) < c < \varphi(u(h_d^-))$. By concavity of g this implies $g'(\varphi(u(h_d^-))) \leq g'(c)$. Hence, $g(\varphi(u(h_d^-))) - g\left(\varphi\left(EU_p^t\right)\right) \geq g'(\varphi(u(h_d^-)))\left(\varphi(u(h_d^-)) - \varphi\left(EU_p^t\right)\right)$. Consequently,

$$\sum_{supp(\mu)} \mu(p_i)\left[g\left(\varphi\left(u(h_d^-)\right)\right) - g\left(\varphi\left(EU_{p_i}^t\right)\right)\right] \geq g'(\hat{\varphi}) \sum_{supp(\mu)} \mu(p_i)\left[\varphi\left(u(h_d^-)\right) - \varphi\left(EU_{p_i}^t\right)\right]$$

Let V_1^j denote the utility of DM1 and V_2^j denote the utility of DM2 under the decision j. The above expression then becomes $V_2^- - V_2^t \geq g'(\hat{\varphi})(V_1^- - V_1^t)$. Because g is increasing, if $V_1^- - V_1^t > 0$, $V_2^- - V_2^t > 0$. In other words, whenever DM1, whose ambiguity aversion is captured by φ, chooses not to treat without prior testing, the more ambiguity averse DM2, whose ambiguity aversion is

captured by an increasing and concave transformation of φ, will also choose not to treat. Together with the continuity of the utility function, this concludes the proof.

Claim 4. The test-treatment threshold (referring to the failure rate) under therapeutic ambiguity aversion is lower than test-treatment threshold under ambiguity neutrality. Assuming that both decision makers share the same beliefs, this means that if DM2 is more ambiguity averse than DM1, DM2 is less inclined to treat without prior testing than DM1 such that

(i) if DM1 decides to test, DM2 will also do so
(ii) if DM1 is indifferent between testing and treatment, DM2 will choose to test first
(iii) there is a (subjective) probability of treatment failure at which DM1 chooses treatment while DM2 decides to test

Proof of Claim 4. $\varphi(EU_p^t) - \varphi(EU_p^+)$ is negative for $p = 0$, positive for $p = 1$, and increases with p. Together with continuity of u and φ, this implies that $\exists p^*$ such that $\varphi(EU_{p^*}^t) = \varphi(EU_{p^*}^+) = \hat{\varphi}$. Let g be an increasing and concave function. By the mean value theorem,$\forall p \in \ supp(\mu), \exists c$ between $\varphi(EU_p^+\ )$ and $\varphi(EU_p^t)$ such that $g(\varphi(EU_p^t)) - g(\varphi(EU_p^+)) = g'(c)(\varphi\ (EU_p^t\ ) - \varphi(EU_p^+))$. For $p < p^*$, $\hat{\varphi} < \varphi(EU_p^t) < c < \varphi(EU_p^+)$. By concavity of g this implies $g'(\hat{\varphi}) \geq\ g'(c)$. Hence, $g(\varphi(EU_p^t)) - g(\varphi(EU_p^+)) \geq\ g'(\hat{\varphi})(\varphi(EU_p^t) - \varphi(EU_p^+))$. For $p > p^*,\ \hat{\varphi}\ > \varphi(EU_p^t) > c > \varphi(EU_p^+)$. By concavity of g this implies $g'(\hat{\varphi}) \leq\ g'(c)$. Hence, \$ $g(\varphi(EU_p^t)) - g(\varphi(EU_p^+)) \geq g'(\hat{\varphi})(\varphi(EU_p^t) - \varphi(EU_p^+))$. Consequently,

$$\sum_{supp(\mu)} \mu(p_i)[g\left(\varphi(EU_{p_i}^t)\right) - g\left(\varphi(EU_{p_i}^+)\right)] \geq g'(\hat{\varphi}) \sum_{supp(\mu)} \mu(p_i)[\varphi(EU_{p_i}^t) - \varphi(EU_{p_i}^+)]$$

Let V_1^j denote the utility of DM1 and V_2^j denote the utility of DM2 under the treatment decision j. The above expression then becomes $V_2^t - V_2^+ \geq\ g'(\hat{\varphi})\backslash left(V_1^t - V_1^+\ )$. Because g is increasing, if $V_1^t - V_1^+ > 0, V_2^t - V_2^+\ > 0$. In other words, whenever DM1, whose ambiguity aversion is captured by φ, chooses to test, the more ambiguity averse DM2, whose ambiguity aversion is captured by an increasing and concave transformation of φ, will also choose to test. Together with the continuity of utility function, this concludes the proof.

A.3 Diagnostic Model Under Unconditional Ambiguity Aversion

In this section, we apply the notion of unconditional ambiguity aversion as studied in Hoy et al. (1) (where ambiguity arises from the uncertainty of the test results from an ex-ante perspective) to our diagnostic ambiguity model and show that the demand for diagnostic testing decreases if DMs are averse to such an ambiguity.

In Hoy et al. (1), the only source of ambiguity is the uncertainty surrounded by the test result, which means that the DM is conditionally ambiguity-neutral (i.e., is indifferent about the ambiguity over the correct distribution of disease probability) but unconditionally ambiguity averse.[1] A conditional

[1] Here, we use the terminology of Nocetti (2) to differentiate between the two types of ambiguity. To our knowledge, this is the only paper that makes this distinction. More precisely, Nocetti defines *conditional ambiguity* as "the uncertainty

ambiguity neutral DM is indifferent to the spread in the expected utilities caused by multiple possible probability distributions, even though they are informed of such multiplicity. They therefore take a weighted average of possible probabilities and use this expected prior to the ex-ante evaluation. In our setting, the weighted expected prior probability of disease is $\bar{p} = \mu\, p_H + (1-\mu)p_L$. The probability of receiving a positive test result can then be written as $\lambda^+ = \bar{p}Se + (1-\bar{p})(1-Sp)$. Similarly, the probability of receiving a negative test result is $\lambda^- = \bar{p}(1-Se) + (1-\bar{p})Sp$. The posterior probabilities of being sick and healthy once a specific test result is received then become

$$\underbrace{p^{sp} = \frac{\bar{p}Se}{\lambda^+}}_{\substack{\text{prob.of being sick} \\ \text{once a positive result} \\ \text{has obtained}}}, \quad \underbrace{p^{hp} = \frac{(1-\bar{p})(1-Sp)}{\lambda^+}}_{\substack{\text{prob.of being healthy} \\ \text{once a positive result} \\ \text{has obtained}}}, \quad \underbrace{p^{sn} = \frac{\bar{p}(1-Se)}{\lambda^-}}_{\substack{\text{prob.of being sick} \\ \text{once a negative result} \\ \text{has obtained}}}, \quad \underbrace{p^{hn} = \frac{(1-\bar{p})Sp}{\lambda^-}}_{\substack{\text{prob.of being healthy} \\ \text{once a negative result} \\ \text{has obtained}}}$$

Suppose φ is a concave function capturing unconditional ambiguity aversion. The expected utility of no treatment for the unconditional ambiguity averse DM is then equal to $\varphi(\bar{p}u(h_d^-) + (1-\bar{p})u(h_h^-))$, which corresponds to $\varphi(\mu\, EU_{p_H}^- + (1-\mu)EU_{p_L}^-)$ in the notation of this paper. Similarly, the expected utility of treatment under unconditional ambiguity aversion is $\varphi(\bar{p}u(h_d^+) + (1-\bar{p})u(h_h^+))$, which corresponds to $\varphi(\mu\, EU_{p_H}^+ + (1-\mu)EU_{p_L}^+)$ in our notation. Finally, the expected utility of testing becomes

$$\lambda^+\, \varphi\,(p^{sp}u(h_d^+) + p^{hp}u(h_h^+)) + \lambda^-\, \varphi\,(p^{sn}u(h_d^-) + p^{hn}u(h_h^-))$$

which is smaller than $\varphi(\mu EU_{p_H}^t + (1-\mu)EU_{p_L}^t)$ by the concavity of φ. Suppose that at $\mu = \mu_A^t$, the (unconditional) ambiguity averse DM is indifferent between testing and no treatment, and at $\mu = \mu_A^{tRx}$, they are indifferent between testing and treatment. This implies the following:

$$\varphi(\mu_A^t\, EU_{p_H}^- + (1-\mu_A^t)EU_{p_L}^-) < \varphi(\mu_A^t\, EU_{p_H}^t + (1-\mu_A^t)EU_{p_L}^t)$$

$$\Rightarrow\ \mu_A^t\, \mathrm{EU}_{p_H}^- + (1-\mu_A^t)\mathrm{EU}_{p_L}^- < \mu_A^t\, \mathrm{EU}_{p_H}^t + (1-\mu_A^t)\mathrm{EU}_{p_L}^t$$

$$\varphi(\mu_A^{tRx}\, EU_{p_H}^+ + (1-\mu_A^{tRx})EU_{p_L}^+) < \varphi(\mu_A^{tRx}\, \mathrm{EU}_{p_H}^t + (1-\mu_A^{tRx})\mathrm{EU}_{p_L}^t)$$

$$\Rightarrow \mu_A^{tRx}\ EU_{p_H}^+ + (1-\mu_A^{tRx})EU_{p_L}^+ < \mu_A^{tRx}\, EU_{p_H}^t + (1-\mu_A^{tRx})EU_{p_L}^t$$

The above equations show that the ambiguity-neutral DM already tests when the ambiguity-averse DM is indifferent (i.e., $\mu_A^t > \mu_N^t$.). Moreover, the ambiguity-neutral DM still tests when the ambiguity-averse DM is indifferent between testing and direct treatment (i.e., $\mu_A^{tRx} < \mu_N^t$). In conclusion, aversion to the ambiguity associated with the test result decreases the demand for diagnostic testing.

A.4 Therapeutic Model Under Unconditional Ambiguity Aversion

We now show that under therapeutic uncertainty, unconditional ambiguity aversion also decreases the demand for diagnostic testing. Assume that the DM uses a weighted expected a priori probability $\bar{p} = \mu\, p_H + (1-\mu)p_L$ of treatment failure. The probability of getting a positive test result can then

---

over the correct distribution of outcomes that remains after a message is received" and *unconditional ambiguity* as "uncertainty over the message which will be received".

be written as $\lambda^+ = \bar{p}Se + (1-\bar{p})(1-Sp)$. Similarly, the probability of getting a negative test result is $\lambda^- = \bar{p}(1-Se) + (1-\ \bar{p})Sp$. The posterior probabilities of failed and successful treatment once a specific test result is received then become

$$\underbrace{p^{fp} = \frac{\bar{p}Se}{\lambda^+}}_{\substack{\text{prob.of treatment failure}\\ \text{once a positive result}\\ \text{has obtained}}}, \quad \underbrace{p^{sp} = \frac{(1-\bar{p})(1-Sp)}{\lambda^+}}_{\substack{\text{prob.of successful treatment}\\ \text{once a positive result}\\ \text{has obtained}}}, \quad \underbrace{p^{fn} = \frac{\bar{p}(1-Se)}{\lambda^-}}_{\substack{\text{prob.of treatment failure}\\ \text{once a negative result}\\ \text{has obtained}}}, \quad \underbrace{p^{sn} = \frac{(1-\bar{p})Sp}{\lambda^-}}_{\substack{\text{prob.of successful treatment}\\ \text{once a negative result}\\ \text{has obtained}}}$$

Suppose φ is a concave function capturing unconditional ambiguity aversion. The expected utility of no treatment for the unconditional ambiguity averse DM is then equal to $\varphi(\ u(h_d^-))$. The expected utility of treatment under unconditional ambiguity aversion is $\varphi\big(\ \bar{p}u\big(h_d^{+f}\big) + (1-\ \bar{p})u(h_d^{+s})\big)$, which corresponds to $\varphi\big(\mu\ EU_{p_H}^+ + (1-\mu\ )EU_{p_L}^+\big)$ in our notation. Finally, the expected utility of testing becomes

$$\lambda^+\ \varphi\ (u(h_d^-\ )) + \lambda^-\ \varphi\ \big(p^{fn}u\big(h_d^{+f}\big) +\ p^{sn}u(h_d^{+s})\big)$$

which is smaller than $\varphi(\mu EU_{p_H}^t) + (1-\mu)EU_{p_L}^t)$ because of the concavity of φ. Suppose that at $\mu = \mu_A^t$, the (unconditional) ambiguity-averse DM is indifferent between testing and no treatment and that at $\mu = \mu_A^{tRx}$, they are indifferent between testing and treatment. This implies the following:

$$\varphi(u(h_d^-)) < \varphi\big(\mu_A^t\ EU_{p_H}^t + (1-\mu_A^t)EU_{p_L}^t\big)$$

$$\Rightarrow\ u(h_d^-) < \mu_A^t\ \mathrm{EU}_{p_H}^{\mathrm{t}} + (1-\mu_A^t)\mathrm{EU}_{p_L}^{\mathrm{t}}$$

$$\varphi\big(\mu_A^{tRx}\ EU_{p_H}^+ + (1-\mu_A^{tRx})EU_{p_L}^+\big) < \varphi\big(\mu_A^{tRx}\ \mathrm{EU}_{p_H}^{\mathrm{t}} + (1-\mu_A^{tRx})\mathrm{EU}_{p_L}^{\mathrm{t}}\big)$$

$$\Rightarrow \mu_A^{tRx}\ \ EU_{p_H}^+ + (1-\mu_A^{tRx})EU_{p_L}^+ < \mu_A^{tRx}\ EU_{p_H}^t + (1-\mu_A^{tRx})EU_{p_L}^t$$

The above equations show that the ambiguity neutral DM still tests when the ambiguity-averse DM is indifferent between testing and no treatment (i.e., $\mu_A^t > \mu_N^t$.). Moreover, the ambiguity-neutral DM starts testing when the ambiguity-averse DM is indifferent between testing and direct treatment i.e., $\mu_A^{tRx} < \mu_N^t$. In conclusion, aversion to the ambiguity associated with the test result decreases the test range.

A.5 References

1. Hoy M, Peter R, Richter A. Take-up for genetic tests and ambiguity. *J Risk Uncertain* (2014) 48:111–133. doi:10.1007/s11166-014-9186-z

2. Nocetti DC. Ambiguity and the value of information revisited. *Geneva Risk Insur Rev* (2018) 43:25–38. doi:10.1057/s10713-018-0025-z
